# Supplementary material for: Expression of Nectin-4 and PD-L1 in Upper Tract Urothelial Carcinoma
Source: Int J Mol Sci. 2020 Jul 29;21(15):5390. doi: 10.3390/ijms21155390 (PMC7432817; doi:10.3390/ijms21155390)
Supplement: Supplementary file 1 [file ijms-21-05390-s001.zip › Supplementary figures.pdf]

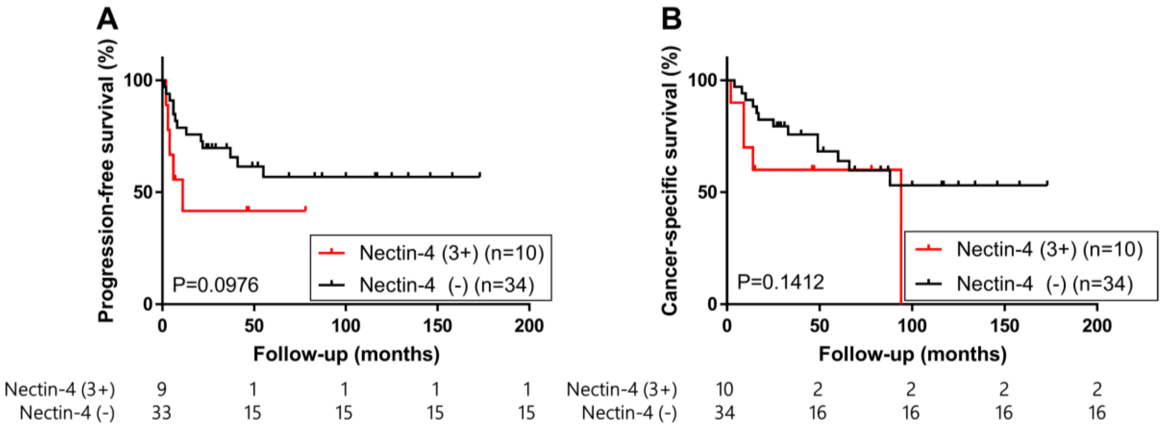

Supplementary Figure S1 Progression-free survival (A) and cancer-specific survival (B) in 99 patients with upper tract urothelial carcinoma (UTUC) based on Nectin-4 expression (0 versus 3+) .

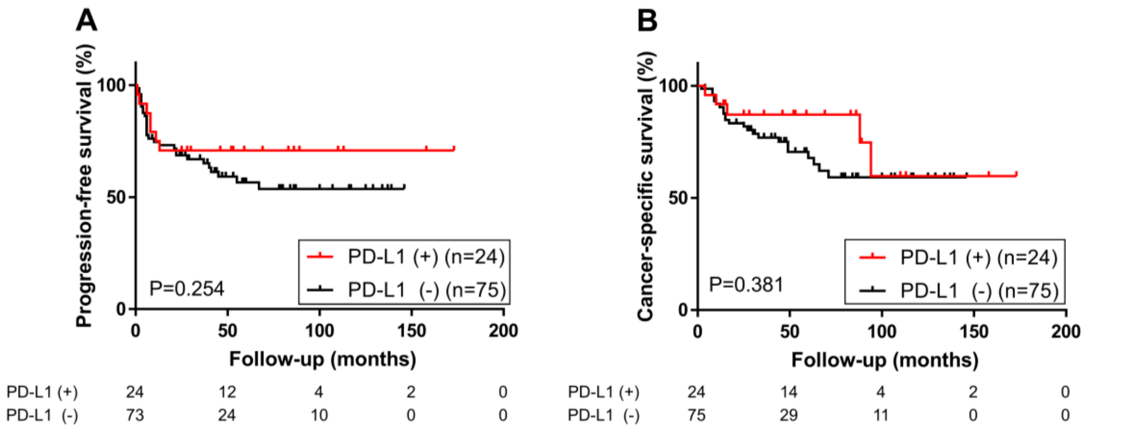

Supplementary Figure S2 Progression-free survival (A) and cancer-specific survival (B) in 99 patients with upper tract urothelial carcinoma (UTUC) based on Programmed Death Ligand 1 (PD-L1) expression.
