# Supplementary material for: Expression of Nectin-4 and PD-L1 in Upper Tract Urothelial Carcinoma
Source: Int J Mol Sci. 2020 Jul 29;21(15):5390. doi: 10.3390/ijms21155390 (PMC7432817; doi:10.3390/ijms21155390)
Supplement: Supplementary file 1 [file ijms-21-05390-s001.zip › Supplementary Table S1.docx]

**Supplementary Table S1.** Association of PD-L1 expression with clinicopathological characteristics of UTUC

| Variable | PD-L1 expression, n (%) | | P-value |
| --- | --- | --- | --- |
|  | Positive | Negative | Positive vs negative |
| Sex |  |  | **0.034** |
| Male | 10 (16.7) | 50 (83.3) |  |
| Female | 14 (35.9) | 25 (64.1) |  |
| Tumour location |  |  | 0.812^a^ |
| Renal pelvis | 11 (24.4) | 34 (75.6) |  |
| Ureter | 11 (22.0) | 39 (78.0) |  |
| Both |  |  |  |
| Tumour grade |  |  | 0.755 |
| Low grade | 4 (26.7) | 11 (73.3) |  |
| High grade | 20 (23.8) | 64 (76.2) |  |
| Pathological T stage |  |  | 1.000^b^ |
| pTa | 5 (26.3) | 14 (73.7) |  |
| pT1 | 4 (22.2) | 14 (77.8) |  |
| pT2 | 1 (12.5) | 7 (87.5) |  |
| pT3 | 11 (22.9) | 37 (77.1) |  |
| pT4 | 3 (50.0) | 3 (50.0) |  |
| Lymphovascular invasion |  |  | 1.000 |
| No | 14 (23.7) | 45 (76.3) |  |
| Yes | 10 (25.0) | 30 (75.0) |  |
| Lymph node metastasis |  |  | 1.000^c^ |
| pN0 | 21 (25.0) | 63 (75.0) |  |
| pN+ | 3 (25.0) | 9 (75.0) |  |
| pNx | 0 (0.0) | 3 (100.0) |  |

a Renal pelvis vs Ureter

b pTa+pT1 vs pT2+pT3+pT4

c pN0 vs pN+
